# fNIRS evidence for enhanced brain activity during task-based Arabic learning in virtual reality

**DOI:** 10.1016/j.ynirp.2026.100358

**Published:** 2026-05-17

**Authors:** Noble Amadi, Wei-Chiang Lin, Noha Elsakka, Melissa Baralt

**Affiliations:** a Florida International University, Department of Biomedical Engineering, United States; b Florida International University, Department of Modern Languages, United States

**Keywords:** SLA (second language acquisition), SL2 (social brain of language learning framework), TBLT (task-based language learning), Virtual reality, AL2 (Arabic as an additional language)

## Abstract

**Significance:**

As one of the world's most widely spoken languages, Arabic holds immense cultural and geopolitical importance, but traditional teaching methods often fall short in preparing learners for real-life interaction. Virtual reality (VR) and task-based language teaching (TBLT) offer promising, immersive alternatives, but the neurobiological mechanisms underlying their effectiveness remain unclear. Understanding these mechanisms can inform second language pedagogy.

**Aim:**

This study investigated whether immersive, task-based Arabic vocabulary learning in VR elicits stronger brain activity and superior learning outcomes than traditional lecture-style instruction.

**Approach:**

Twelve English-dominant adults with no prior Arabic knowledge were randomly assigned to VR or traditional learning groups. Participants completed receptive and productive vocabulary pre- and posttests while cortical activity was recorded using functional near-infrared spectroscopy (fNIRS). fNIRS signals were preprocessed and compared across groups.

**Results:**

Both groups showed significant pre-to-post gains in receptive vocabulary scores. However, the VR group exhibited significantly greater improvements in productive performance (p = 0.002). fNIRS analyses revealed consistently higher hemodynamic variability in prefrontal (Broca's area), parietal and superior temporal (Wernicke's area) regions for The VR group during learning and subsequent tests, indicating greater brain activity.

**Conclusions:**

VR-mediated TBLT enhances cortical activation and productive Arabic performance compared with traditional instruction, offering neurobiological evidence for immersive technologies in language learning.

## 1 Introduction

The global demand for Arabic language instruction has never been more urgent. Spoken by over 300 million people across 22 countries, Arabic is increasingly vital in international diplomacy, business, healthcare, and education. In the United States, Arabic is the fastest-growing language, with a 581% increase in home use since 1980 (Moslimani, 2023). Yet, despite this demand, Arabic remains underrepresented in classrooms, with fewer than 1% of U.S. college students enrolled in Arabic courses (Looney et al., 2023). These challenges are shaped in part by Arabic's linguistic complexity, including its diglossic structure, with Modern Standard Arabic (MSA) coexisting alongside diverse dialects, and its extended learning trajectory, requiring over 2000 instructional hours for native English speakers to achieve proficiency. (Foreign Service Institute (FSI)) As such, learners often face a disconnect between formal classroom instruction and real-world communicative needs (Albirini, 2015; Elsakka, 2023; Huntley, 2024).

### 1.1 Task-based and immersive approaches

To address these challenges, Arabic as an Additional Language (AL2) pedagogy has shifted away from traditionally grammar-focused instruction toward learner-centered, task-based approaches. Task-Based Language Teaching (TBLT) positions real-world tasks (not grammatical forms) as the foundation for instruction and assessment (Ellis, 2003; Long, 2015; East, 2012). When anchored in Task-Based Needs Analysis (TBNA), TBLT ensures alignment with learners’ communicative demands across both MSA and colloquial varieties (Elsakka, 2023; Gilabert et al., 2025). Alongside this pedagogical evolution, immersive technologies such as Virtual Reality (VR) have gained traction. Unlike traditional (Trad) classroom settings, VR enables embodied, socially grounded interaction in context-rich environments, thereby engaging memory, visuospatial, and sensorimotor systems critical to language acquisition (Legault et al., 2019). The Social Brain of Language Learning (SL2) framework further posits that language learning is fundamentally social and embodied, predicting that immersive settings like VR will enhance brain activity and learning outcomes (Li et al., 2020a).

A growing body of research has examined the role of virtual reality (VR) in second language vocabulary learning, with findings converging on several key patterns. First, both low- and high-immersion VR environments can support vocabulary acquisition by providing contextualized, multimodal input that integrates visual, auditory, and experiential cues, thereby strengthening encoding and recall (Kaplan‐Rakowski et al., 2025; Chen et al., 2022). However, the degree of immersion alone does not determine learning outcomes. Studies show that highly immersive VR does not consistently outperform lower-immersion or traditional conditions on immediate vocabulary assessments, although advantages are more frequently observed in long-term retention (Kaplan‐Rakowski et al., 2025; Schnitzer et al., 2025; Sun et al., 2025). These findings suggest that the benefits of VR emerge over time, particularly when learners engage with language in meaningful, embodied contexts. Second, increasing evidence highlights the role of interaction and embodiment. Active engagement with virtual objects and environments, especially when paired with sensorimotor involvement, has been shown to enhance vocabulary learning by linking lexical items to perceptual and motor experiences (Kaplan‐Rakowski et al., 2025; Sun et al., 2025). Third, learning outcomes in VR are highly dependent on instructional design. Task structure, level of interactivity, and opportunities for meaningful communication appear to play a more decisive role than immersion alone, with poorly designed VR environments offering limited benefits (Sun et al., 2025; Chen et al., 2022). Finally, although VR research has demonstrated consistent gains in engagement, motivation, and receptive vocabulary outcomes, relatively little work has examined its impact on productive language use (Sun et al., 2025). This gap is particularly notable given that productive skills are central to achieving proficiency and may be especially sensitive to the embodied and interactive affordances of VR.

Building on this emerging body of work, there remains a clear need for VR-based language learning environments that integrate task-based pedagogy, meaningful social interaction, and a focus on productive language use. To address this gap, Virtual Tabadul (Baralt et al., 2022) was developed as the first VR-based, task-driven Arabic–English virtual exchange program. Grounded in Task-Based Language Teaching and intercultural citizenship models (O'Dowd, 2020), it pairs U.S. students learning Arabic with peers from the Middle East and North Africa (MENA) learning English to complete authentic communicative tasks. Over the semester, pairs meet via Zoom or Google Meet multiple times and use smartphones with Google Cardboard viewers to enter low-immersion (LiVR) spherical-video/360° VR scenes, such as a Moroccan market, an Algerian heritage site, or a Miami classroom, where audiovisual clues are embedded to support interaction. Each exchange follows a structured pre-task, during-task, and post-task sequence: learners first review key language and cultural elements, then collaborate to complete goal-oriented tasks in the VR environment and finally engage in reflection and application activities. Designed to be low-cost and accessible, the program leverages widely available tools (e.g., Canvas, Zoom, A-Frame) while fostering language learning as well as intercultural awareness, social connection, and global citizenship. Importantly, learners participate as co-constructors of meaning, contributing cultural knowledge and engaging in tasks that reflect real-world communicative needs. Recent behavioral findings indicate that learners in Virtual Tabadul outperform peers in traditional classroom settings on productive language tasks, though the neurobiological mechanisms underlying these advantages remain unexplored.

### 1.2 Neuroimaging and language learning

To understand the mechanisms underlying these behavioral advantages, it is necessary to examine how different instructional contexts shape brain activity during language learning. To address this gap, understanding how the brain responds to different learning contexts and rewires its networks during adult second language acquisition (SLA) has become a central focus in applied linguistics and cognitive neuroscience. Advances in neuroimaging, particularly functional Magnetic Resonance Imaging (fMRI), Electroencephalography (EEG), and functional Near-Infrared Spectroscopy (fNIRS) have provided valuable insights into how distinct instructional contexts shape brain plasticity, connectivity, and cognitive load. Each modality offers distinct strengths for capturing different dimensions of language learning, including spatial organization, temporal dynamics, and task-based cortical engagement.

#### 1.2.1 fMRI

Among these approaches, functional magnetic resonance imaging (fMRI) has provided foundational insights into how large-scale brain networks support second language (L2) learning. Resting-state studies demonstrate that increases in proficiency are associated with shifts in functional connectivity among prefrontal, temporal, and parietal regions, reflecting experience-dependent reorganization of language and control networks (). Task-based and longitudinal fMRI research further suggests that early stages of learning are characterized by widespread recruitment of these regions, with activity patterns gradually consolidating into more efficient and specialized configurations as learners gain experience. (Gkintoni et al., 2025), (Yang et al., 2015) Similar transitions from localized activation profiles to more distributed connectivity patterns have been observed in broader skill-learning paradigms (Bertolero et al., 2018).

Importantly, however, this literature does not imply that greater activation per se indexes superior learning or proficiency. Rather, fMRI findings emphasize stage-dependent changes in network organization and coordination, highlighting how early learning engages flexible and distributed systems that later become more economical. In bilingual contexts, both resting-state and task-based fMRI studies show that language control and maintenance rely on adaptive connectivity changes across cortical networks (Isel et al., 2019). Collectively, these findings demonstrate that L2 acquisition involves dynamic neural adaptation marked by both stability and plasticity, but they are primarily concerned with network-level reconfiguration, rather than fine-grained task-evoked cortical dynamics within a single learning session ().

#### 1.2.2 EEG

To capture these rapid, within-task neural dynamics, neuroimaging approaches with high temporal resolution are required. Electroencephalography (EEG) offers complementary insight into second language acquisition by capturing the rapid temporal dynamics of neural processing. EEG studies have shown that even brief exposure to a novel language can elicit measurable neural changes, with early learning engaging prefrontal and temporoparietal systems within minutes of instruction (Steber et al., 2021). These findings underscore the brain's capacity for rapid adaptation and its sensitivity to learning demands.

Beyond amplitude-based responses, EEG research has increasingly highlighted the relevance of neural variability and temporal dynamics as markers of active cognitive engagement. For example, EEG and EEG–fNIRS studies have revealed sex-dependent and strategy-specific differences in syntactic learning, suggesting that early learning may be supported by distinct processing modes rather than uniform increases in activation (Sugiura et al., 2018). Such work reinforces the view that learning-related neural changes are not solely reflected in stronger responses, but also in the dynamic modulation of neural activity over time.

While EEG excels in temporal precision, its spatial limitations constrain the ability to localize cortical sources during complex, naturalistic learning tasks. As such, EEG findings motivate, but do not directly resolve questions about how moment-to-moment neural engagement unfolds across cortical regions during immersive, interactive language learning, particularly in ecologically valid, movement-rich learning environments such as virtual reality.

#### 1.2.3 fNIRS

To address these limitations and enable the study of language learning in naturalistic, interactive contexts, functional near-infrared spectroscopy (fNIRS) has emerged as a powerful modality for studying second language acquisition in ecologically valid settings, particularly where movement, speech production, and interaction are integral to the task. Like fMRI, fNIRS measures task-related hemodynamic responses through changes in oxygenated (HbO) and deoxygenated (HbR) hemoglobin concentrations. Unlike fMRI, however, fNIRS allows for naturalistic, movement-tolerant data collection, making it well suited for investigations of real-world learning environments, including virtual reality (VR).

Rather than focusing exclusively on network connectivity or long-term neural reorganization, much fNIRS research in SLA has examined task-evoked cortical engagement and its sensitivity to cognitive, social, and instructional factors. Reviews highlight the growing use of fNIRS to study bilingual cognition and educational practices (Pliatsikas, 2024).-^25^ Empirical findings demonstrate that fNIRS captures meaningful differences in cortical engagement associated with multilingual switching (Farrukh et al., 2025), instructional style and teacher–learner synchrony (Zhang et al., 2024), and socially framed versus non-social instruction (Christensen, 2025).

Emerging fNIRS literature suggests that within-task cortical engagement as indexed by hemodynamic variability (standard deviation) can provide behaviorally relevant information (Halliday et al., 2018). Variability in hemodynamic signals has been associated with attentional engagement, cognitive load, and flexible recruitment of neural resources, particularly in complex or interactive tasks (Halliday et al., 2018; Armbruster-Genç et al., 2016). This perspective aligns with broader neuroimaging work indicating that variability reflects the brain's dynamic operating range rather than noise (Garrett et al., 2010).

Building on these pedagogical and neuroimaging insights, the present study employs fNIRS to examine how task-based language instruction in VR modulates cortical engagement during early-stage Arabic vocabulary learning, in comparison to traditional online instruction. While fMRI research has elucidated principles of network reorganization ()– (Yang et al., 2015) and EEG has captured rapid temporal dynamics of early learning (Steber et al., 2021), (Sugiura et al., 2018) fNIRS offers a critical bridge between controlled neuroscience and ecological validity by enabling continuous measurement of cortical dynamics during naturalistic learning. (Pliatsikas, 2024), (Christensen, 2025) By situating Arabic language learning within a VR-based task-based language teaching framework, this study provides a neurobiologically informed evaluation of immersive instructional contexts (Virtual Tabadul) without presupposing connectivity reorganization or long-term neural reconfiguration.

### 1.3 Aims and research hypotheses

The aim of the present study is to investigate how immersive, task-based instruction in virtual reality influences both behavioral and neural outcomes in Arabic language learning, compared to a traditional lecture-based online format. By combining behavioral assessments with fNIRS, this study seeks to identify the cognitive and neurobiological mechanisms underlying the potential advantages of VR-based learning.

Based on prior evidence that immersive, socially grounded environments enhance brain activity in second language acquisition, we propose the following hypotheses. First, participants in the VR condition will demonstrate greater gains in both receptive (comprehension) and productive (oral production) vocabulary performance than those in the traditional condition. Second, VR-based language learning will be associated with greater task-evoked cortical engagement, reflected in increased neurovascular variability in prefrontal and language-related regions during the learning phase. Third, these neural differences will persist into the post-learning receptive and productive tasks, aligning with the observed behavioral advantages.

In doing so, the study provides the first neurobiologically informed evaluation of Virtual Tabadul and extends the Social Brain of Language Learning (SL2) framework to immersive Arabic instruction, offering new insight into how digital technologies shape language learning processes.

## 2 Methodology

### 2.1 Participant recruitment

In this exploratory study, healthy young adults with no prior knowledge of Arabic were recruited through word of mouth and email announcements. To maintain a homogeneous sample and control for linguistic background effects, eligibility was restricted to native English speakers. Twelve participants were enrolled for this preliminary study; many also reported speaking or having been exposed to Spanish at home. This study was reviewed and approved by the Florida International University Institutional Review Board (FIU IRB) on April 25, 2022, under protocol number IRB-21-0503. All procedures involving human participants were conducted in compliance with the institutional guidelines and the ethical standards. Written informed consent was obtained from all participants prior to participation. All procedures were completed in a single session and upon completion, participants received $20 in cash as compensation.

### 2.2 Study protocol

**Fig. 1:**
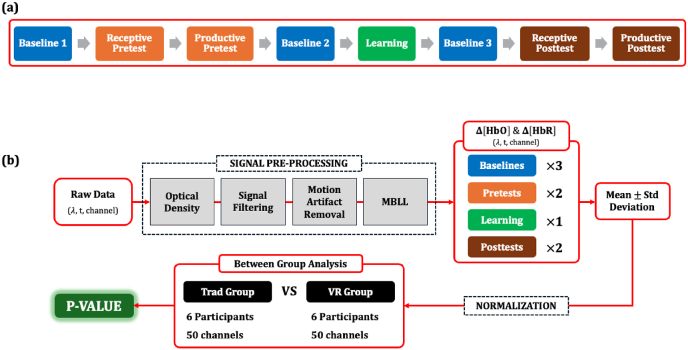
Block diagrams of (a) the study protocol and (b) the fNIRS data analysis pipeline.

The block diagram in Fig. 1 outlines the study protocol. Twelve participants (9 males, 3 females; M = 21.25 ± 2.01 years) were randomly assigned to one of two conditions: the virtual reality (VR) learning group (n = 6) or the traditional (Trad) learning group (n = 6). Upon arrival, participants were fitted with an fNIRS cap, and an initial baseline recording was collected. During this baseline phase (∼2 min), participants sat quietly with their eyes closed and were instructed to relax and clear their minds. Following the baseline, participants completed two language pretests in sequence: first a receptive vocabulary pretest and then a productive vocabulary pretest.

Next, participants engaged in their assigned learning session (VR or Trad). Additional baseline recordings were conducted immediately before and after the learning session (second and third baselines, respectively) to capture resting-state neural activity for comparison. After completing the learning activity, participants performed the receptive and productive posttests in the same order as the pretests. Throughout the entire procedure, cortical activity was continuously monitored using the fNIRS system to track brain activity across all phases of the study.

### 2.3 Arabic language learning

Two distinct strategies for learning Arabic were developed and implemented in this study. One mimicked traditional classroom learning (Trad group) while the other offered an interactive learning experience with VR technology (VR group).

Participants assigned to the Trad group viewed a 12-min recorded lecture on a laptop. During the lecture, the instructor wrote each English word or phrase on a virtual whiteboard, demonstrated its pronunciation in Arabic, and encouraged participants to repeat each word aloud after her. This approach aimed to resemble a conventional grammar-based classroom experience typical of Arabic language instruction (Elsakka, 2023).

Participants assigned to the VR group began with a brief setup and orientation. They accessed a secure link on their smartphones, enabled audio, disabled notifications, and placed their devices in Google Cardboard headsets. A VR coordinator connected via Zoom helped participants orient themselves in the VR space and confirmed they could see all four critical landmarks that led to key greeting phrases in both English and Arabic. After completing the orientation, participants explored a series of five interactive “clues” within the 3D classroom. For example, the first clue involved locating shared cups of colada coffee on a desk. Once they found the cups, participants received an audio-visual lesson that included close-up imagery and bilingual narration, teaching them the words “cup” and “cups” in Arabic. Each subsequent clue followed a similar pattern, systematically introducing the same target vocabulary. The entire VR session lasted approximately 15 min, including setup and minor technical troubleshooting.

### 2.4 Receptive test

The purpose of the receptive test was to assess each participant's understanding of the target Arabic vocabulary after one learning session. In the receptive test, each participant completed 15 multiple-choice questions using Qualtrics on a laptop. Each question presented an English word or phrase (e.g., “Hello”) and asked the participant to select the correct Arabic transliteration (“marḥaban”) from a list of six options. Each item included six response options (one correct answer and five distractors), reducing the probability of correct responses due to chance (≈16.7%) while supporting item discrimination. Distractor options consisted of real Arabic words selected to provide plausible alternatives, with some items sharing semantic or morphological features with the target forms (e.g., greetings or possessive structures), thereby requiring participants to rely on learned lexical knowledge rather than superficial recognition, consistent with principles of receptive knowledge assessment (Gass and Mackey, 2007). All responses were recorded and graded automatically by the Qualtrics system. A 15-s time limit was imposed for each question to reduce the likelihood of extended deliberation or random responding, while encouraging participants to rely on more immediate lexical knowledge, consistent with prior research on time constraints and automaticity in second language processing (DeKeyser, 2007; Towell et al., 1996). Participants were not able to return to prior items, further limiting the use of test-taking strategies such as answer revision or process of elimination across items (Baralt, 2013). Two versions of this test were administered, once before the learning began and once after. Internal consistency reliability was assessed using Cronbach's alpha and indicated acceptable reliability for both versions (pretest α = 0.82; posttest α = 0.80).

### 2.5 Productive test

The productive test sought to assess the participant's ability to produce Arabic language after one learning session. This measure was developed by the researchers to align directly with the specific vocabulary and linguistic features introduced during the instructional phase. During the productive test, each participant was shown a set of 20 printed notecards, each featuring an English term or phrase. The task was administered individually by an Arabic language instructor who is a native speaker with over ten years of experience teaching Arabic as an Additional Language in U.S. university settings, ensuring consistent delivery across participants. The participants were instructed to either say the corresponding Arabic word aloud or state “I don't know” if they were unsure.

The set of items was constructed to reflect the instructional targets and included five classroom-related nouns (e.g., “professor”), their plural forms (e.g., “professors”), and four basic communicative phrases (e.g., “How are you?“). In addition, five distractor items (untrained words, such as “music”) were included to assess the specificity of learning, and one ungraded bonus item was incorporated to probe participants' ability to generalize the Arabic pluralization rule (Gass and Mackey, 2007). Item order was randomized across participants to minimize potential order effects. A digital voice recorder was used to capture all responses for subsequent transcription and scoring. All responses were transcribed and scored using a predefined answer key, with binary coding (correct/incorrect) based on target lexical forms. Phonologically recognizable approximations of target items were accepted, whereas incorrect or unrelated lexical forms were marked as incorrect. To establish scoring reliability, 40% of the responses were independently coded by a second trained rater. Inter-rater reliability was high (Cohen's κ = 0.92), indicating strong agreement between coders. Any discrepancies were subsequently discussed and resolved through consensus. As with the receptive test, this measure was administered twice: once before the learning session and once after.

To limit potential priming and carryover effects across testing phases, several design features were incorporated. These included the use of parallel versions of receptive test at pre- and posttest, a fixed administration order in which the productive test preceded the receptive test, and the absence of feedback during testing. Together, these procedures were intended to ensure that posttest performance reflected learning rather than prior exposure to test items.

## 3 Data analysis

### 3.1 Analysis of the receptive and productive results

To evaluate learning following the session, within-group comparisons of pretest and posttest scores were conducted separately for receptive and productive measures. Normality of score distributions was first assessed using the Shapiro–Wilk test to determine whether the datasets satisfied the assumption of normality. Based on these results, paired t-tests (for normally distributed data) or Wilcoxon signed-rank tests (for non-normal data) were used to evaluate pre-to-post changes within each group.

To quantify learning gains, an improvement (gain) score was calculated for each participant by subtracting the pretest score from the corresponding posttest score for each test type. Between-group comparisons were then conducted to assess whether learning outcomes differed between instructional modalities. Depending on the distribution of the data, two-tailed t-tests or Mann–Whitney U tests were used to compare gain scores between the VR and Traditional groups for both receptive and productive measures. Given that the receptive test consisted of 15 multiple-choice questions with seven response options, chance-level performance corresponds to approximately 2.14 correct responses. One-sample tests were conducted to compare observed pretest scores against this chance level. In addition, to evaluate baseline comparability between the VR and Traditional groups, equivalence testing was performed using the two one-sided tests (TOST) procedure. This analysis was applied to both receptive and productive pretest scores to determine whether group differences fell within predefined equivalence bounds.

### 3.2 fNIRS data analysis

#### 3.2.1 fNIRS system

**Fig. 2:**
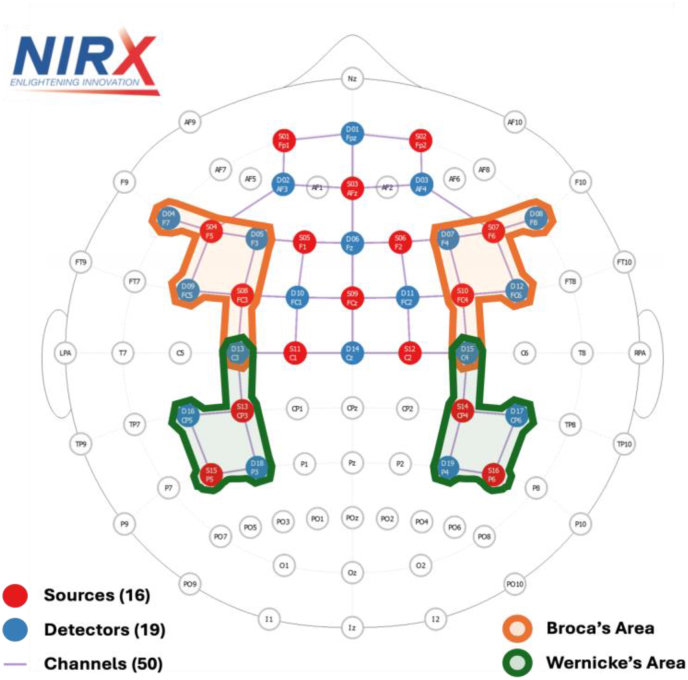
A top‐down view of the NIRx fNIRS cap used in this study.

In addition to the receptive and productive behavioral tests, fNIRS signals were collected from each participant using the NIRx NIRScout system (NIRx Medical Technologies). This system consists of sixteen fiber-optic light sources and nineteen detectors embedded in a head cap, as shown in Fig. 2. This configuration produced fifty source-detector pairs, referred to as “channels,” which spanned the frontal and temporal cortices. Notably, twenty-two channels were positioned over regions corresponding to Broca's area (inferior frontal gyrus) and Wernicke's area (superior temporal gyrus), both critical regions for language processing (Zhang et al., 2023; Čeko et al., 2024; Wani, 2024). Channel placement was guided by standard optode configuration protocols to approximate coverage of these cortical regions. The source-detector separation of each channel was approximately 3 to 3.5 cm, ensuring sufficient investigation depth while maintaining a high signal-to-noise ratio. Two illumination wavelengths in the NIR range (760 nm & 850 nm) were used to monitor fluctuations in the concentrations of oxyhemoglobin (HbO) and deoxyhemoglobin (HbR) within the tissue volume being investigated. The responses from all 50 channels were sampled at a rate of 3.9 Hz during each recording session. Before starting the experimental protocol, a cap was fitted on each participant, and the optical coupling of each channel was checked and calibrated using the NIRStar acquisition software from NIRx Medical Technologies to ensure high-quality signals.

#### 3.2.2 Preprocessing

Raw fNIRS signals were acquired as a function of wavelength (λ), time (t), and channel (ch); they were denoted as V(λ,t,ch). Prior to any statistical analysis, V(λ,t,ch) underwent a multi-stage preprocessing pipeline, as illustrated in Fig. 1b, designed to condition the signals and minimize motion artifacts in them. Specifically, V(λ,t,ch) was first transformed into optical density (OD) using the following equation:

$$OD\left(\lambda ,t,ch\right)=-\log\;\;\log\;\left(\frac{V\left(\lambda ,t,ch\right)}{mean\left(V\left(\lambda ,t,ch\right)\right)}\right)$$

The optical density signals (OD(λ,t,ch)), were then filtered using a band-pass filter with a passband of 0.2 Hz to 0.8 Hz [1] targeting higher-frequency hemodynamic fluctuations that have been shown to contain meaningful neural information beyond traditional low-frequency components (Lu et al., 2017; Xu et al., 2023; Lei et al., 2020). The Temporal Derivative Distribution Repair algorithm (Fishburn et al., 2019) was then applied to the filtered OD(λ,t,ch) signals to reduce motion-induced distortions. Finally, the conditioned OD(λ,t,ch) signals were converted into changes in oxy-hemoglobin and deoxy-hemoglobin concentrations, denoted as Δ[HbO](t,ch) and Δ[HbR](t,ch), respectively, using the modified Beer–Lambert law.

#### 3.2.3 Feature extraction

The processed Δ[HbO](t,ch) and Δ[HbR](t,ch) (hemodynamic signal type) were segmented according to the study activities (act) depicted in Fig. 1a. This resulted in eight subsections for each signal - three Baselines (act=bl1−bl3), two receptive tests (act=rt), two productive tests (act= pt), and one Learning session (act=ln), which were denoted as Δ[HbO](t,act,ch) and Δ[HbR](t,act,ch). For each subsection, the mean and standard deviation were calculated on a per-channel basis. These features were extracted across all 50 fNIRS channels and for all participants; they were denoted as Mean(i,act,ch,Hb) and SD(i,act,ch,Hb) where i is the participant number and Hb represents the hemodynamic signal type.

#### 3.2.4 Statistical analyses

Recorded fNIRS data was then statistically analyzed to compare brain activity during and after the acquisition of new Arabic vocabulary between the VR and Trad groups.

The first statistical analysis aimed to determine if VR group brain activity during learning elicits distinct patterns, as measured by Δ[HbO](t,ln,ch) and Δ[HbR](t,ln,ch), compared to traditional group brain activity during learning. Prior to the statistical comparison, baseline normalization was performed to reduce inter-subject variability and hence isolate the learning-related brain activity. That is:

(1) $$MeanNorm\left(i,\ln,ch,Hb\right)=\frac{Mean\left(i,\ln,ch,Hb\right)}{Mean\left(i,bl2,ch,Hb\right)}$$

(2) $$SDNorm\left(i,\ln,ch,Hb\right)=\frac{SD\left(i,\ln,ch,Hb\right)}{SD\left(i,bl2,ch,Hb\right)}$$

where bl2 indicates the feature that is extracted from the baseline recording prior to the learning session (Fig. 1a). These baseline-normalized features from participants in the VR learning condition were statistically compared with those from the Traditional learning condition on a channel-by-channel basis. Prior to between-group analysis, the distribution of feature values for each channel was assessed for normality using the Shapiro–Wilk test. For channels in which the normality assumption was satisfied, t-tests were used, and when the assumption of normality was violated, Mann–Whitney U tests were employed instead. The significance level was set at α = 0.05. To control for multiple comparisons arising from the 50 channel-wise tests performed for each condition, Benjamini–Hochberg false discovery rate (FDR) correction was applied with q = 0.05. The resulting adjusted p-values were denoted as pMean(ln,ch,Hb) and pSD(ln,ch,Hb), respectively.

The second statistical analysis aimed to elucidate how the learning method influences brain activity during the post-learning receptive and productive tests. This investigation sought to explain the differences in learning efficiency observed in the receptive and productive tests. First, the mean and standard deviation features from the pretest and posttest segments were normalized to those from their corresponding baselines. Subsequently, to further isolate learning induced changes specific to the post receptive and productive tests, a second normalization step was applied. That is:

$$MeanNorm2\left(i,act,ch,Hb\right)=\frac{MeanNorm_{post}\left(i,act,ch,Hb\right)}{MeanNorm_{pre}\left(i,act,ch,Hb\right)}$$

$$SDNorm2\left(i,act,ch,Hb\right)=\frac{SDNorm_{post}\left(i,act,ch,Hb\right)}{SDNorm_{pre}\left(i,act,ch,Hb\right)}$$

where act ∈ {rt, pt} denotes either the receptive or productive test, and the subscripts ‘pre’ and ‘post’ indicate features extracted from the pretest and posttest segments, respectively. The normalized features from the participants of the VR group were statistically compared to those of the traditional group. Again, this included a normality check which was assessed using the Shapiro–Wilk test. For channels in which the normality assumption was satisfied, t-tests were used, and when the assumption of normality was violated, Mann–Whitney U tests were employed instead. The significance level was set at α = 0.05. To control for multiple comparisons arising from the 50 channel-wise tests performed for each condition, Benjamini–Hochberg false discovery rate (FDR) correction was applied with q = 0.05. The resulting p-values were denoted as pMean (rt,ch,Hb) and pSD (rt,ch,Hb) for the receptive test and pMean (pt,ch,Hb) and pSD (pt,ch,Hb) for the productive test.

Both the mean and standard deviation of the hemodynamic signals were analyzed, however, the standard deviation was selected as the target fNIRS feature since the TDDR algorithm used for motion artifact correction normalized the signal baselines, bringing mean values close to zero and thereby obscuring group-level differences. Raw p-values and FDR adjusted p-values are reported in the supplementary document.

## 4 Results section

### 4.1 Behavioral outcomes

**Fig. 3:**
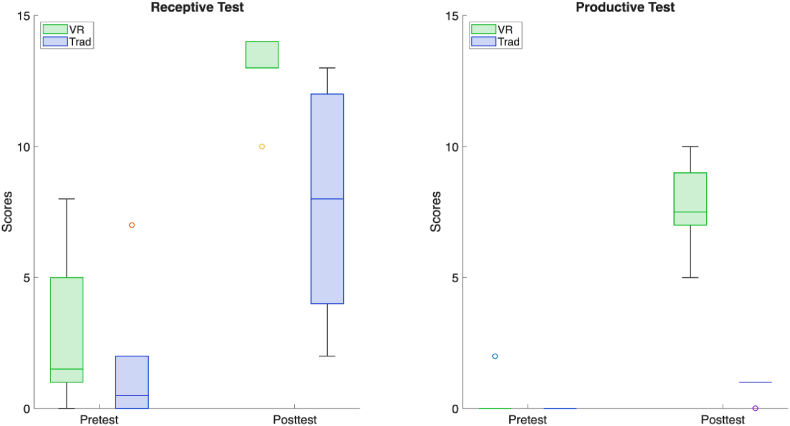
Receptive (left) and productive (right) test scores from the VR and Trad groups.

The receptive and productive test scores for all participants are presented in Table 1. The results are also shown in boxplots in Fig. 3 Pretest scores in both the VR and Traditional groups were low, indicating minimal prior knowledge of Arabic. A chance-level analysis of the receptive pretest showed that neither the VR group (*p* = 1.0000) nor the Traditional group (*p* = 0.4375) performed reliably above chance. Productive pretest scores were uniformly at or near zero across participants, further confirming the absence of prior productive knowledge. No statistically significant differences were observed between groups at pretest for either the receptive (*p* = 0.368) or productive (*p* = 1.000) measures. To further evaluate baseline comparability, equivalence testing using the two one-sided tests (TOST) procedure was conducted. Results indicated statistical equivalence between groups for the productive pretest. However, equivalence was not statistically confirmed for the receptive pretest, indicating that baseline comparability for this measure remains inconclusive.

**Table 1:** Mean ± standard deviation (SD) of receptive and productive test scores for the virtual reality (VR) and traditional learning groups. Pretest and posttest scores are presented alongside improvement scores (posttest − pretest), illustrating the magnitude of learning gains following a single instructional session.

Measure	VR (Mean ± SD)	Traditional (Mean ± SD)
Receptive Pretest	2.83 ± 3.06	1.67 ± 2.73
Receptive Posttest	12.83 ± 1.47	7.83 ± 4.36
Receptive Improvement	10.00 ± 3.10	6.17 ± 4.96
Productive Pretest	0.33 ± 0.82	0.00 ± 0.00
Productive Posttest	7.67 ± 1.75	0.83 ± 0.41
Productive Improvement	7.33 ± 1.63	0.83 ± 0.41

Following the learning session, both groups demonstrated significant improvements from pretest to posttest on both receptive and productive measures (see Table 2) (see Table 3).

**Table 2:** Results of within-group comparisons between pretest and posttest scores for receptive and productive measures in both VR and traditional groups. Statistical significance was assessed using either the Wilcoxon signed-rank test or paired *t*-test, depending on normality assumptions.

Group	Measure	PValue	Test Used	Interpretation
VR	Receptive	0.031	Wilcoxon signed-rank test	Significant pre-post change
Traditional	Receptive	0.031	Wilcoxon signed-rank test	Significant pre-post change
VR	Productive	0.031	Wilcoxon signed-rank test	Significant pre-post change
Traditional	Productive	0.004	Paired *t*-test	Significant pre-post change

**Table 3:** Results of between-group comparisons (VR vs. Traditional) for pretest scores and learning gains (improvement = posttest − pretest) in receptive and productive measures. Statistical significance was assessed using the Mann–Whitney *U* test.

Measure	PValue	Test Used	Interpretation
Receptive Pretest	0.368	Mann-Whitney *U* test	No significant between-group difference
Productive Pretest	1.000	Mann-Whitney *U* test	No significant between-group difference
Receptive Improvement	0.193	Mann-Whitney *U* test	No significant between-group difference
Productive Improvement	0.002	Mann-Whitney *U* test	Significant between-group difference

To measure the degree of improvement in each test type, the differences between posttest scores and their corresponding pretest scores were calculated and referred to as ‘Improvement’. For the receptive test, the improvement in the VR group was greater than in the Trad group, but this difference was not statistically significant (p = 0.193), indicating comparable gains in Arabic language comprehension across both learning methods. In contrast, the improvement in the productive test was significantly higher in the VR group compared to the Trad group (p = 0.002), suggesting that immersive VR learning offered an advantage for productive language capacity, specifically, the ability to retrieve and generate Arabic forms.

### 4.2 fNIRS hemodynamic findings

**Fig. 4:**
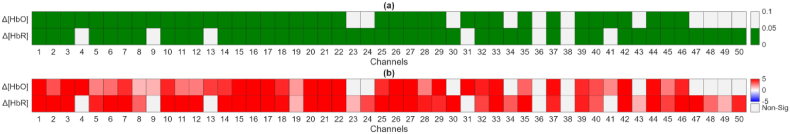
Statistical analysis of learning effects across channels. (a) pSD (ln,ch,Hb) map: channels shown in green indicate a statistically significant effect (p < 0.05). (b) ΔSD map: a gradient color bar was used to encode the value of ΔSD, with color transitions reflecting changes in magnitude.

The target fNIRS feature, SDNorm (i,ln,ch,Hb), from both groups did not meet the assumption of normality, as indicated by the Shapiro-Wilk test result (p > 0.05). Consequently, the Mann-Whitney *U* test was employed to assess differences between the two learning modalities. The result of this statistical comparison, pSD (ln,ch,Hb), as presented in Fig. 4a, indicated a statistical difference in brain activity between the VR and Trad groups and across most measurement channels. This suggests that the immersive VR environment elicited higher hemodynamic variability across the majority of the measured channels compared to traditional instructional methods during vocabulary learning.

To elucidate how SDNorm (i,ln,ch,Hb) varied between the two groups, the difference in their average SDNorm (i,ln,ch,Hb) was analyzed for each channel and hemoglobin type. Specifically, the following was calculated:

(3) $$\Delta SD\left(\ln,ch,Hb\right)={\underline{SDNorm}}_{VR}\left(\ln,ch,Hb\right)-{\underline{SDNorm}}_{TRAD}\left(\ln,ch,Hb\right)$$

where ${\underline{SDNorm}}_{VR}\left(\ln,ch,Hb\right)$ and ${\underline{SDNorm}}_{TRAD}\left(\ln,ch,Hb\right)$ denote the average of SDNorm (i,ln,ch,Hb) from the VR and the Trad groups, respectively. For channels with pSD (ln,ch,Hb) less than 0.05, their corresponding ΔSD (ln,ch,Hb) values were shown in Fig. 4b. In this ΔSD map, nearly all 50 channels are red (i.e., ΔSD (ln,ch,Hb) > 0) for both Δ[HbO] and Δ[HbR]. This striking trend indicates that consistently higher hemodynamic variability was exhibited by the VR group throughout the frontal, parietal and superior temporal regions, including Broca's and Wernicke's areas.

#### 4.2.1 Receptive test

**Fig. 5:**
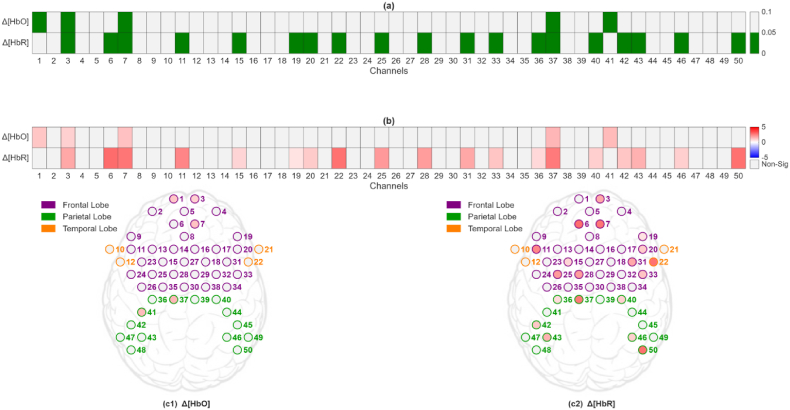
Changes in brain activity during the receptive test caused by learning. (a) pSD (ln,ch,Hb) map: channels shown in green indicate a statistically significant effect (p < 0.05). (b) ΔSD map: a gradient color bar was used to encode the value of ΔSD, with color transitions reflecting changes in magnitude. (c) topographic ΔSD maps.

To examine how learning-related neural changes were expressed during task performance, we analyzed cortical activity during the post-learning receptive test. The effects of different learning styles on alterations in brain activity during the receptive test were assessed using SDNorm2 (i,rt,ch,Hb). Since the SDNorm2 (i,rt,ch,Hb) from both the VR and Trad groups was found not to follow a normal distribution (Shapiro-Wilk test, p > 0.05), the Mann-Whitney *U* test was again utilized for the between-group comparison. The results of this comparison, pSD (rt,ch,Hb) are graphically presented in Fig. 5a, and they clearly indicate that a statistically significant difference in SDNorm2 (i,rt,ch,Hb) exists between the two learning modalities across several channels.

To elucidate how SDNorm2 (i,rt,ch,Hb) varied between the two groups, the difference in average SDNorm2 (i,rt,ch,Hb) between the two groups was analyzed using the strategy proposed in Eq-3. ΔSD (rt,ch,Hb) for pSD (rt,ch,Hb) < 0.05 is graphically presented in Fig. 5b. As shown in this ΔSD map, the majority of channels exhibit positive values, indicating that the VR group maintained significantly greater hemodynamic variability across the cortex, even during the passive, multiple-choice receptive test. Inspecting the topographic ΔSD (rt,ch,Hb) maps for each hemoglobin type (Fig. 5c) reveals more nuance. For ΔSD (rt,ch,HbO), light red hues primarily appear over the frontal and parietal regions, signifying modestly increased oxy-hemoglobin fluctuations for the VR group, while the superior temporal regions are less active. In contrast, the ΔSD (rt,ch,HbR) map shows more intense red channels in the frontal and parietal regions, the superior temporal region still remains less active. This indicates that the VR group showed higher deoxy-hemoglobin variability and thus greater metabolic demand in both frontal executive and posterior language cortices during Arabic comprehension.

#### 4.2.2 Productive test

**Fig. 6:**
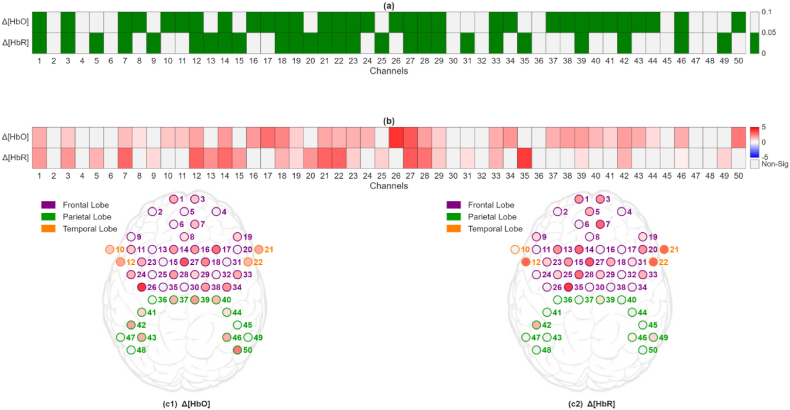
Changes in brain activity during the productive test caused by learning. (a) pSD (ln,ch,Hb) map: channels shown in green indicate a statistically significant effect (p < 0.05). (b) ΔSD map: a gradient color bar was used to encode the value of ΔSD, with color transitions reflecting changes in magnitude. (c) topographic ΔSD maps.

To determine whether learning-related neural differences extended to conditions requiring active language production, we analyzed cortical activity during the post-learning productive test. The effects of different learning types on alterations in brain activity during the productive test were assessed using SDNorm2 (i,pt,ch,Hb). Data from both the VR and Trad groups were evaluated for normality using the Shapiro-Wilk test, which indicated that the distributions did not meet the assumption of normality (p > 0.05). As a result, the Mann-Whitney *U* test was employed to compare SDNorm2 values between the two groups. The outcomes of this statistical comparison, denoted as pSD (pt,ch,Hb), are shown in Fig. 6a. These results demonstrated that statistically significant differences in SDNorm2 (i,pt,ch,Hb) were observed between the VR and Trad groups in frontal, parietal and superior temporal regions. This finding suggests that the mode of learning had a measurable impact on brain activity alterations during the productive phase of the language task.

To examine how SDNorm2 (i,pt,ch,Hb) varied between two groups, ΔSD (pt,ch,Hb) was calculated from their average SDNorm2 values for each fNIRS channel using the method described in Eq-3. Fig. 6b is a graphical display of ΔSD (pt,ch,Hb) for those fNIRS channels with pSD (rt,ch,Hb) less than 0.05. It was observed that the majority of the ΔSD (pt,ch,Hb) was positive, which is similar to what was observed in the ΔSD analysis for the receptive test. Furthermore, this ΔSD map is characterized by a deeper red saturation compared to the maps generated from ΔSD (rt,ch,Hb), as shown in Fig. 6b. The topographic ΔSD maps provide a spatially resolved depiction of this effect, revealing that the brain regions (frontal, parietal, and superior temporal) exhibited pronounced increases in both Δ[HbO] and Δ[HbR] signal variability (Fig. 6c). This shows that the effects of VR learning elicited stronger brain activity in language areas during speech production.

## 5 Discussion

### 5.1 Overview of findings

This study examined whether immersive, task-based language learning in virtual reality leads to different behavioral and neural outcomes compared to traditional instruction. The behavioral findings revealed that both the VR and Traditional groups improved from pretest to posttest. For the receptive measure, the VR group achieved higher overall scores than the Traditional group; however, the difference in improvement between groups was not statistically significant (p = 0.193). In contrast, the productive measure revealed a clear divergence: the VR group demonstrated substantially greater gains than the Traditional group (p = 0.002), whose improvement was minimal.

The neural findings, derived from fNIRS analysis, provide additional insight into the mechanisms underlying these behavioral results. During the learning session, between-group comparisons revealed that the VR group exhibited higher Δ[HbO] and Δ[HbR] variability (SDNorm) across many channels relative to the Traditional group. This pattern reflects that VR learning elicited stronger and broader brain activity than traditional learning during the learning phase. To examine how these learning-related differences were expressed during subsequent task performance, we introduced the SDNorm2 index, which captures relative changes in normalized Δ[HbO] and Δ[HbR] variability from pretest to posttest. For the receptive test, both groups demonstrated increases in SDNorm2 (SDNorm2 > 1), reflecting learning-related modulation of cortical dynamics during posttest performance. Between groups, the VR group exhibited moderately higher SDNorm2 values, particularly in frontal and parietal regions, with larger effects observed in Δ[HbR] signals. For the productive test, both groups again showed elevated SDNorm2 values following learning; however, the VR group demonstrated substantially greater increases than the Traditional group. These effects were widespread across frontal, parietal, and superior temporal regions and were evident in both Δ[HbO] and Δ[HbR] signals. They also demonstrate that VR enhanced brain activity and learning efficiency more effectively than traditional instruction, particularly in productive contexts. Importantly, these neural patterns align with the behavioral findings, as the largest between-group differences were observed in the productive condition. This convergence suggests that immersive VR learning more strongly modulates cortical dynamics under conditions requiring active retrieval and production of language, rather than recognition alone.

### 5.2 Language networks and plasticity

These findings should not be interpreted as direct evidence of neural reorganization or long-term plasticity. Rather, they indicate that even brief immersive VR exposure can give rise to measurable changes in task-related cortical engagement, reflecting how neural systems respond dynamically to newly learned material. Prior neuroimaging research has shown that early stages of language learning engage widespread cortical regions, with patterns of activation and coordination evolving as proficiency increases (Yang et al., 2015; Gkintoni et al., 2025). Longitudinal fMRI studies suggest that these early stages are characterized by flexible and distributed recruitment, which later consolidates into more specialized network configurations with experience (Yang et al., 2015). Similarly, EEG and fNIRS studies have demonstrated that short learning sessions on the order of minutes can elicit detectable neurophysiological changes associated with learning and task engagement (Steber et al., 2021). Together, this literature supports the idea that limited L2 exposure can modulate neural engagement during task performance, even in the absence of measurable network-level reconfiguration.

Within this context, the stronger SDNorm2 observed in the VR group relative to the Trad group is neurobiologically informative insofar as it suggests greater learning-related modulation of cortical dynamics, rather than stronger or broader activation in an absolute sense. Intra-session hemodynamic variability, quantified here as the standard deviation (SD) of the fNIRS signal, was selected as the primary neural index because it captures moment-to-moment fluctuations in cortical engagement over extended task periods. Prior neuroimaging work has demonstrated that signal variability (SD) reflects the brain's dynamic operating range, indexing attentional engagement, cognitive demand, and flexible recruitment of neural resources, and that this variability carries independent and behaviorally relevant information, particularly during cognitive tasks (Halliday et al., 2018; Garrett et al., 2010; Armbruster-Genç et al., 2016). Thus, the elevated SDNorm2 associated with VR learning likely reflects more dynamic and sustained engagement of language-relevant cortical regions, particularly during post-learning tasks.

Crucially, the present study does not measure functional connectivity or neural reconfiguration directly. Therefore, claims regarding accelerated reorganization or shifts from localized to distributed connectivity cannot be made on the basis of these data. A more accurate and conservative interpretation is that the VR condition elicited greater channel-level hemodynamic variability, and this occurred alongside larger behavioral improvements. Greater channel-level variability in the VR group also reflects heightened engagement driven by embodied interaction, social contextualization, and active task demands inherent to immersive environments (Li et al., 2020a). This interpretation is consistent with the Social Brain of Language Learning (SL2) framework and Task-Based Language Teaching (TBLT) principles, which emphasize engagement, interaction, and meaningful goal-directed activity as drivers of effective language learning (Ellis, 2003; Long, 2015; Li et al., 2020a). Notably, this effect was most pronounced during the productive test, where participants were required to actively retrieve and produce language and where the VR group also demonstrated the largest behavioral gains.

#### 5.2.1 Receptive test

The receptive test examined participants’ ability to recognize Arabic transliterations corresponding to English words, engaging processes of visual recognition, lexical retrieval, and decision-making. Analysis of SDNorm2 of Δ[HbO] and Δ[HbR] revealed that both groups exhibited neural modulation associated with learning, though the VR group displayed higher SDNorm2 and hence positive ΔSD across key cortical regions associated with language acquisition, including the frontal (dorsolateral prefrontal cortex – DLPFC, ventrolateral prefrontal cortex–VLPFC), parietal (inferior parietal lobe–IPL), and temporal (superior temporal gyrus– STG) regions.

It is known that the DLPFC is responsible for executive control, attentional regulation, and working memory during lexical decision-making (Hertrich et al., 2021; Thothathiri et al., 2012). Increased ΔSD (rt,DLPFC,HbR) in this region represents higher metabolic activity, indicating that the DLPFC of The VR group was more actively engaged in sustaining attention toward novel linguistic stimuli and managing cognitive load during recognition of unfamiliar word forms. In other words, the VR group was able to engage more executive processes to maintain attentional focus and accuracy during word recognition than the Trad group.

VLPFC is known to be responsible for lexical retrieval, morphosyntactic structuring, and verbal working memory maintenance (Thothathiri et al., 2012; Badre et al., 2007). The moderate ΔSD (rt,VLPFC,HbO) and strong ΔSD (rt,VLPFC,HbR) reflect that the VLPFC of the VR group was strongly recruited to mediate linguistic control specifically by selecting the correct transliteration among competing options and managing morphosyntactic associations between word forms. This interpretation concurs with evidence suggesting that the VLPFC plays a key role in controlled lexical selection and the inhibition of competing representations during early stages of L2 learning (Indefrey et al., 2004; Badre et al., 2007).

IPL is known for its function in phonological decoding and in mapping orthographic representations to phonological and semantic networks (Bzdok et al., 2016; Coslett et al., 2018). The moderate ΔSD (rt,VLPFC,HbO) and ΔSD (rt,VLPFC,HbR) observed in this study reflects a more active integration between visual and articulatory representations of the VR group, crucial for establishing cross-modal associations between written Arabic forms and their phonological equivalents.

Finally, existing literature shows that STG is responsible for lexical-semantic access and comprehension and plays a critical role in processing phonological and semantic information (Hickok and Poeppel, 2007). However, since the receptive test has no auditory demands and our fNIRS channels did not cover the superior temporal region, we do not have strong evidence to explain how different learning styles influence its engagement in the receptive test.

#### 5.2.2 Productive test

The productive test required participants to recall and verbally produce Arabic words from English-equivalent cues, engaging lexical retrieval, phonological encoding, motor planning, and speech articulation. Analysis of SDNorm2 of Δ[HbO] and Δ[HbR] revealed widespread changes in brain activity across frontal, parietal, and superior temporal regions. These changes were especially pronounced in the VR group, as evident by ΔSD (Fig. 6). This phenomenon suggests that the VR group activated a more integrated and dynamic language network than The Trad group during speech production in the productive test.

The strong ΔSDs in VLPFC reflect more elevated neural effort directed toward selecting appropriate lexical items while suppressing competing alternatives in the VR group, an executive function essential for fluent language production. The inferior frontal gyrus (IFG) plays a critical role in phonological encoding, syntactic integration, and the controlled retrieval of semantic information during word generation (Indefrey et al., 2004). The strong ΔSDs in IFG observed in this study demonstrate a stronger engagement of this region of the VR group in transforming lexical and syntactic information into articulatory output.

Similar characteristics of ΔSDs have also been observed in the middle frontal gyrus (MFG) and pre-supplementary motor area (pre-SMA) of the VR group. These two areas are critical in the productive test because (1) MFG is involved in working memory and executive control necessary for planning and maintaining linguistic content during utterance construction (Hagoort, 2013), and (2) pre-SMA is known to play a role in sequencing and timing articulatory motor programs and inhibiting competing motor actions (Hertrich et al., 2021; Alario et al., 2006). These findings together indicate that frontal regions, especially VLPFC and IFG, of the VR group are more activated to orchestrate top-down control processes that facilitate fluent speech production and grammatical precision.

Moderate ΔSDs were also noticed in the parietal cortical region, especially in the superior parietal lobe (SPL) and inferior parietal lobe (IPL). SPL is known to be involved in phonological working memory and auditory–motor integration (Paulesu et al., 1993; Golestani and Zatorre, 2009). IPL, on the other hand, is responsible for semantic integration and sensorimotor mapping for articulation (Bzdok et al., 2016). Therefore, our fNIRS results imply that The VR group can activate both parietal regions more efficiently to synchronize phonological planning with motor execution during speech production of the productive test.

Lastly, elevated SDNorm2 and large ΔSDs are also observed in STG, which is well known to monitor auditory feedback and perform phonological encoding during overt speech (Hickok and Poeppel, 2007; Schwartz et al., 2009). These results confirm participants’ reliance on auditory self-monitoring mechanisms to ensure phonetic accuracy and fluency during spoken output, and the VR group was able to activate these regions more efficiently during the productive test.

### 5.3 Limitations and future directions

While this study provides novel insights into the neural mechanisms underlying VR-based Arabic language learning, several methodological and interpretive limitations should be acknowledged. The most significant constraint concerns the small sample size (n = 12; six participants per group), which limits the generalizability and statistical robustness of the findings. Although the trends observed across behavioral and fNIRS measures were consistent, replication with a larger and more diverse cohort is necessary to confirm the observed effects and reduce susceptibility to individual variability.

The study employed a single-session experimental design, which effectively captured short-term cortical modulation but did not allow for an assessment of long-term neural adaptation. As such, it remains unclear whether the relative changes in Δ[HbO] and Δ[HbR] variability observed in this study persist beyond immediate post-test measures or consolidate into durable language learning effects. Future longitudinal investigations should therefore examine the trajectory of cortical reorganization and retention over multiple VR-based learning sessions.

One limitation of the present study is the potential influence of a novelty effect associated with the use of virtual reality (VR). Participants in the VR condition were exposed to a new and immersive technological environment, which may have increased cortical engagement independent of language learning processes. While such novelty-related cognitive demands may contribute to heightened neural activity during the learning phase, they are unlikely to fully account for the observed differences in post-learning receptive and productive tasks, during which participants were no longer interacting with the VR environment. Notably, the VR group demonstrated greater improvements in productive performance alongside increased cortical variability during learning, suggesting that the observed neural differences are at least partially related to learning-specific processes rather than novelty alone.

A further limitation pertains to the scope of the learning module. The instructional content primarily consisted of greetings and simple Arabic phrases, which, although suitable for beginning-level learners and, arguably, a proof-of-concept design, restricts the generalizability of the results to more advanced linguistic constructs such as morphosyntactic forms, discourse-level comprehension, and pragmatic language use. Expanding the complexity and linguistic range of VR modules in future research would allow for examination of how immersive learning influences higher-order language functions.

Importantly, the fNIRS recordings obtained during the active learning phase were prone to motion artifacts, particularly within the VR group. Natural movements, such as gesturing or adjusting head position while the fNIRS cap was on introduced mechanical noise into the hemodynamic signal. Although motion-correction preprocessing was applied, residual artifacts may have affected the precision of the hemodynamic signals we acquired. Future studies should adopt a system that can accommodate large head movements without introducing a lot of artifacts to the signal.

Another methodological limitation involves the analytical scope of the fNIRS data. This study did not perform spatial or temporal correlation analyses; therefore, it was not possible to examine the functional connectivity and interactions among cortical regions. Without network-level analysis, the current findings cannot fully describe how activity propagates between regions such as the IFG, SPL, and STG, or whether activation in one region drives responses in another. Future research should incorporate approaches to characterize the dynamic inter-regional communication underlying second language processing.

To advance this research, future work should focus on several directions: (1) recruiting larger and demographically diverse samples to enhance statistical power; (2) implementing longitudinal multi-session VR interventions to evaluate sustained neuroplastic changes; (3) incorporating additional control conditions, such as non-learning VR exposure or repeated VR sessions, to better isolate the effects of novelty from language-specific learning mechanisms. (4) expanding linguistic stimuli to include linguistic forms that are not just lexical (5) integrating multimodal neuroimaging (e.g., fNIRS–EEG or fNIRS–fMRI); to improve temporal and spatial resolution; and (6) examining how immersive VR modules can be systematically incorporated into Arabic language curricula.

### 5.4 Conclusion

This study provides one of the first neurobiologically informed examinations of VR-based Arabic language learning using fNIRS, offering new insight into how immersive instructional contexts shape both behavioral performance and task-evoked cortical dynamics during early stages of second language acquisition. Both the VR and traditional (Trad) groups engaged language-relevant cortical regions during receptive and productive tasks; however, the VR group consistently exhibited greater learning- and test-related modulation of cortical activity, particularly during productive tasks that required active retrieval, recall, and speech production.

During the receptive task, VR-related differences were modest and localized primarily to frontal, parietal, and superior temporal regions. These relatively small effects likely reflect the multiple-choice format of the receptive assessment, which permits recognition-based strategies and may reduce cognitive and linguistic demands. In contrast, the productive task revealed robust and spatially extensive VR > Traditional differences across a fronto-parietal-temporal network encompassing regions implicated in lexical retrieval, phonological planning, working memory, and articulatory sequencing. These neural patterns closely mirrored the behavioral findings, wherein VR participants demonstrated significantly greater gains in productive language performance relative to their Traditional counterparts.

Importantly, the neural effects observed in this study should be interpreted in terms of task-evoked cortical dynamics rather than neural reorganization or long-term plasticity. The primary fNIRS metric (channel-level hemodynamic variability) indexes moment-to-moment fluctuations in neural engagement during continuous task execution. From our study we observed that the VR condition elicited greater channel-level hemodynamic variability, coinciding with better behavioral improvements, particularly in productive language tasks. Thus, our findings link learning-induced changes in cortical engagement, measured as posttest-relative-to-pretest, with performance increments.

Together, these results indicate that VR-based instruction enhances second language acquisition, as reflected in both behavioral gains and neuroimaging. The fNIRS measurements revealed learning-induced increases in task-evoked cortical engagement (posttest relative to pretest) that are linked to improved performance, particularly for productive language use. From an applied perspective, this study demonstrates that fNIRS is a powerful tool for capturing short-term, task-evoked neural dynamics in ecologically valid learning contexts. More broadly, the findings underscore the pedagogical potential of VR as a complementary instructional modality for second language learning, particularly for fostering engagement and performance in expressive language use, while emphasizing the importance of theoretically grounded and methodologically cautious interpretations of neuroimaging data in educational research. Finally, this study contributes to the growing evidence base for Virtual Tabadul, a VR-based Arabic-English virtual exchange program that connects US and MENA-region students across borders, by providing converging neurobiological and behavioral support for its effectiveness in advancing language proficiency through immersive, task-based interaction.

## Disclosures

The authors declare that no relevant financial or potential other conflicts of interest exist.

## CRediT authorship contribution statement

**Noble Amadi:** Formal analysis, Methodology, Software, Visualization, Writing – original draft, Writing – review & editing. **Wei-Chiang Lin:** Conceptualization, Formal analysis, Methodology, Resources, Supervision, Writing – original draft, Writing – review & editing. **Noha Elsakka:** Conceptualization, Data curation, Investigation, Methodology, Validation. **Melissa Baralt:** Funding acquisition, Project administration, Supervision, Writing – review & editing.

## Declaration of competing interest

The authors declare the following financial interests/personal relationships which may be considered as potential competing interests: Melissa Baralt, Ph.D. reports financial support was provided by J. Christopher Stevens Virtual Exchange Initiative. If there are other authors, they declare that they have no known competing financial interests or personal relationships that could have appeared to influence the work reported in this paper.

## Data Availability

The data and analysis scripts that support the findings of this study are openly available in the GitHub repository: NOBLE1264/fNIRS-Arabic-Language-Learning. The repository includes all MATLAB code used for preprocessing, feature extraction, and statistical analysis, as well as representative anonymized fNIRS datasets for VR and Trad groups.

## References

[bib1] Alario F.X., Chainay H., Lehericy S., Cohen L. (2006). The role of the supplementary motor area (SMA) in word production. Brain Res..

[bib2] Albirini Abdulkafi (2015).

[bib3] Armbruster-Genç Diana JN., Ueltzhöffer Kai, Fiebach Christian J. (2016). Brain signal variability differentially affects cognitive flexibility and cognitive stability. J. Neurosci..

[bib4] Badre David, Anthony D., Wagner (2007). Left ventrolateral prefrontal cortex and the cognitive control of memory. Neuropsychologia.

[bib5] Baralt M. (2013). The impact of cognitive complexity on feedback efficacy during online versus face-to-face interactive tasks. Stud. Sec. Lang. Acquis..

[bib6] Baralt Melissa (2022). Virtual tabadul: creating language-learning community through virtual reality. J. Int. Stud..

[bib8] Bertolero Maxwell A. (2018). The network architecture of cognitive skills. Nat. Neurosci..

[bib9] Bzdok D., Hartwigsen G., Reid A., Laird A.R., Fox P.T., Eickhoff S.B. (2016). Left inferior parietal lobe engagement in social cognition and language. Neurosci. Biobehav. Rev..

[bib11] Chen Bing, Wang Yunqing, Wang Lianghui (2022). The effects of virtual reality-assisted language learning: a meta-analysis. Sustainability.

[bib12] Christensen Taylor (2025).

[bib13] Coslett H.B., Schwartz M.F., Vallar G., Coslett H.B. (2018).

[bib14] DeKeyser R.M. (2007).

[bib15] East Martin (2012).

[bib10] Čeko M., Hirshfield L., Doherty E. (2024). Cortical cognitive processing during reading captured using functional-near infrared spectroscopy. Sci. Rep..

[bib16] Ellis Rod (2003).

[bib17] Elsakka Noha (2023).

[bib18] Farrukh Faiza (2025).

[bib19] Fishburn Frank A. (2019). Temporal derivative distribution repair (TDDR): a motion correction method for fNIRS. Neuroimage.

[bib20] Foreign Service Institute (FSI). “Foreign Language Training.” U.S. Department of State, n.d.

[bib21] Garrett Douglas D. (2010). Blood oxygen level-dependent signal variability is more than just noise. J. Neurosci..

[bib22] Gass S.M., Mackey A. (2007).

[bib23] Gilabert Roger, Malicka Malgorzata (2025).

[bib24] Gkintoni Evgenia (2025). Brain-Inspired Multisensory Learning: A Systematic Review of Neuroplasticity and Cognitive Outcomes in Adult Multicultural and Second Language Acquisition. Biomimetics.

[bib25] Golestani N., Zatorre R.J. (2009). Individual differences in the acquisition of second language phonology. Brain Lang..

[bib26] Hagoort P. (2013). MUC (memory, unification, control) and beyond. Front. Psychol..

[bib27] Halliday Drew WR. (2018). Mean and variability in functional brain activations differentially predict executive function in older adults: an investigation employing functional near-infrared spectroscopy. Neurophotonics.

[bib28] Hertrich I., Dietrich S., Blum C., Ackermann H. (2021). The role of the dorsolateral prefrontal cortex for speech and language processing. Front. Hum. Neurosci..

[bib29] Hickok G., Poeppel D. (2007). The cortical organization of speech processing. Nat. Rev. Neurosci..

[bib31] Huntley Elizabeth (2024). Does studying multiple sociolinguistic varieties of a second language impact learning outcomes? Investigating the simultaneous acquisition of vocabulary in both standard and Egyptian Arabic. Crit. Multiling. Stud..

[bib32] Indefrey Peter, Levelt Willem JM. (2004). The spatial and temporal signatures of word production components. Cognition.

[bib33] Isel Frédéric (2019). Neuroplasticity, network connectivity and language processing across the lifespan. Brain and Cognition.

[bib35] Kaplan‐Rakowski Regina, Thrasher Tricia (2025). The impact of high‐immersion virtual reality and interactivity on vocabulary learning. Br. J. Educ. Technol..

[bib36] Legault Jennifer (2019). Immersive virtual reality as an effective tool for second language vocabulary learning. Languages.

[bib37] Lei Miaomei (2020). Using a data-driven approach to estimate second-language proficiency from brain activation: a functional near-infrared spectroscopy study. Front. Neurosci..

[bib38] Li Peng, Jeong Hyun (2020). The social brain of language: grounding second language learning in social interaction. npj Sci. Learn..

[bib41] Long Michael H. (2015).

[bib42] Looney Dennis, Lusin Natalia (2023).

[bib43] Lu Feng-Mei (2017). Optical mapping of the dominant frequency of brain signal oscillations in motor systems. Sci. Rep..

[bib44] Moslimani M. (2023).

[bib46] O'Dowd Robert (2020). A transnational model of intercultural citizenship for foreign language education. Lang. Teach..

[bib47] Paulesu E., Frith C.D., Frackowiak R.S. (1993). The neural correlates of the verbal component of working memory. Nature.

[bib48] Pliatsikas Christos (2024). Examining functional Near-Infrared Spectroscopy as a tool to study brain function in bilinguals. Frontiers in language sciences.

[bib49] Schnitzer Benjamin Lukas (2025). Proceedings of the Extended Abstracts of the CHI Conference on Human Factors in Computing Systems.

[bib50] Schwartz M.F., Kimberg D.Y., Walker G.M., Faseyitan O., Brecher A., Dell G.S., Coslett H.B. (2009). Anterior temporal involvement in semantic word retrieval: voxel-based lesion–symptom mapping evidence from aphasia. Brain.

[bib52] Steber Sylvia (2021). Neural correlates of implicit pseudoword learning: an EEG-fNIRS study. PLoS One.

[bib54] Sugiura Michiru (2018). Adolescent sex differences in syntactic rule learning: an ERP-fNIRS study. Front. Hum. Neurosci..

[bib55] Sun Lu, Song Xiacheng (2025). The effectiveness of virtual reality for K-12 foreign language learning: a systematic review of recent randomized controlled trials. Front. Psychol..

[bib56] Thothathiri M., Kimberg D.Y., Schwartz M.F. (2012). The neural basis of reversible sentence comprehension: evidence from voxel-based lesion symptom mapping in aphasia. J. Cognit. Neurosci..

[bib57] Towell J.N., Hawkins R., Bazergui N. (1996). The development of fluency in advanced learners of French. Appl. Linguist..

[bib59] Wani P.D. (2024). From sound to meaning: navigating wernicke's area in language processing. Cureus.

[bib60] Xu Gongcheng (2023). Test-retest reliability of fNIRS in resting-state cortical activity and brain network assessment in stroke patients. Biomed. Opt. Express.

[bib61] Yang Jiun-Yeu (2015). Neural adaptation during vocabulary learning: a longitudinal fMRI study. Neuropsychologia.

[bib62] Zhang H., Guo Z., Chen F. (2023). Annual International Conference of the IEEE Engineering in Medicine and Biology Society. IEEE Engineering in Medicine and Biology Society. Annual International Conference.

[bib63] Zhang Juan (2024). Bridging Stories and Science: An fNIRS-based hyperscanning investigation into child learning in STEM. Neuroimage.

