# Supplementary material for: fNIRS evidence for enhanced brain activity during task-based Arabic learning in virtual reality
Source: Neuroimage Rep. 2026 May 17;6(2):100358. doi: 10.1016/j.ynirp.2026.100358 (PMC13197770; doi:10.1016/j.ynirp.2026.100358)
Supplement: Multimedia component 1 [file mmc1.docx]

**Normality Assessment of Behavioral Measures Using the Shapiro–Wilk Test**

| **Measure** |  | **VR (P-Value)** | **VR (Decision)** |  | **Trad (P-Value)** | **Trad (Decision)** |
| --- | --- | --- | --- | --- | --- | --- |
| Receptive Pretest |  | 0.0036 | Non-normal |  | 0.0309 | Non-normal |
| Receptive Posttest |  | 0.0232 | Non-normal |  | 0.0000 | Non-normal |
| Receptive Improvement |  | 0.0001 | Non-normal |  | 0.0051 | Non-normal |
| Productive Pretest |  | 0.0992 | Normal |  | NaN | NaN |
| Productive Posttest |  | 0.0000 | Non-normal |  | 0.0992 | Normal |
| Productive Improvement |  | 0.0019 | Non-normal |  | 0.0992 | Normal |

Results of the Shapiro–Wilk normality test applied to behavioral data from the virtual reality (VR) and traditional (Trad) groups across receptive and productive measures. For each condition (pretest, posttest, and improvement), the table reports the corresponding p-value and the resulting normality classification. A significance threshold of α = 0.05 was used, where p > 0.05 indicates that the data does not significantly deviate from a normal distribution (classified as “Normal”), and p ≤ 0.05 indicates a significant deviation from normality (classified as “Non-normal”).

For receptive measures, both VR and traditional groups exhibited non-normal distributions across pretest, posttest, and improvement scores. In contrast, productive measures showed mixed results: the VR group demonstrated normality only in the pretest condition, while posttest and improvement scores were non-normally distributed. The traditional group exhibited normal distributions for productive posttest and improvement scores, while the productive pretest condition could not be evaluated due to zero variance in the data, resulting in undefined (NaN) p-values.

Overall, the predominance of non-normal distributions across conditions supports the use of nonparametric statistical tests (e.g., Wilcoxon signed-rank and Mann–Whitney U tests) for most comparisons, with parametric tests applied selectively where normality assumptions were satisfied.

**FNIRS RESULTS**

**
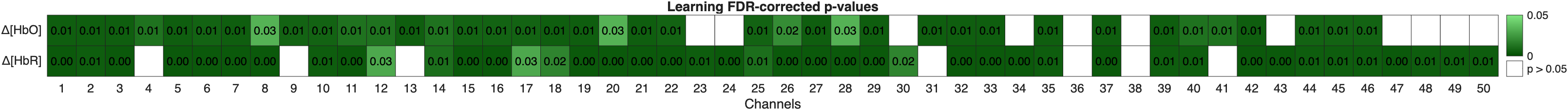

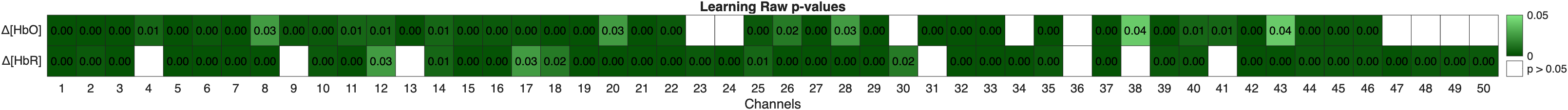
Channel-wise comparison of raw and FDR-corrected p-values for the Learning condition.**

The top panel displays the raw p-values, while the bottom panel shows the Benjamini–Hochberg false discovery rate (FDR)-corrected p-values (q-values) for between-group comparisons (VR vs. Traditional) across all 50 fNIRS channels. Results are presented for both Δ[HbO] (upper row) and Δ[HbR] (lower row).

Each column represents an individual channel, with color intensity indicating statistical significance (darker green = lower p/q-values). Channels with values exceeding the significance threshold (p or q ≥ 0.05) are shown in light gray, and missing channels are displayed in white.

In the raw analysis, a large proportion of channels were identified as significant (HbO: 41/50 channels; HbR: 43/50 channels). Following FDR correction, a substantial number of channels remained significant (HbO: 39/50; HbR: 43/50), indicating that the majority of observed effects are robust to multiple-comparison correction.

Importantly, the minimal reduction in significant channels after FDR correction demonstrates that the observed group differences are not driven by inflated Type I error due to multiple testing.


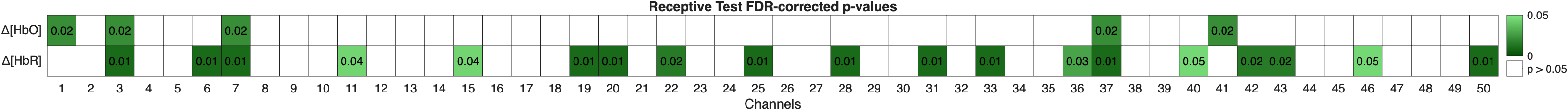
**
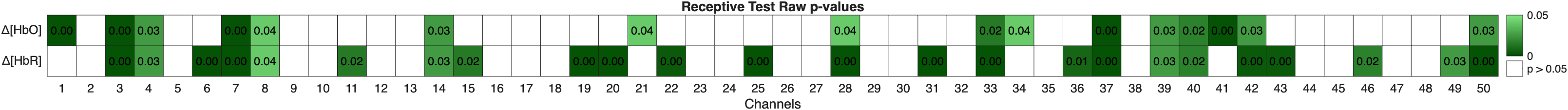
Channel-wise comparison of raw and FDR-corrected p-values for the Receptive Test condition.**

The top panel displays the raw p-values, while the bottom panel shows the Benjamini–Hochberg false discovery rate (FDR)-corrected p-values (q-values) for between-group comparisons (VR vs. Traditional) across all 50 fNIRS channels during the receptive posttest. Results are presented for Δ[HbO] (upper row) and Δ[HbR] (lower row).

Each column corresponds to an individual channel, with darker green indicating stronger statistical significance (lower p- or q-values). Channels with values exceeding the significance threshold (p or q ≥ 0.05) are shown in light gray and missing or excluded channels are displayed in white.

In the raw analysis, several channels exhibited significant group differences (HbO: 16/50; HbR: 24/50). However, after applying FDR correction, the number of significant channels was substantially reduced for HbO (5/50), while a larger subset remained significant for HbR (19/50). This reduction highlights that some of the raw HbO effects may reflect inflated Type I error.


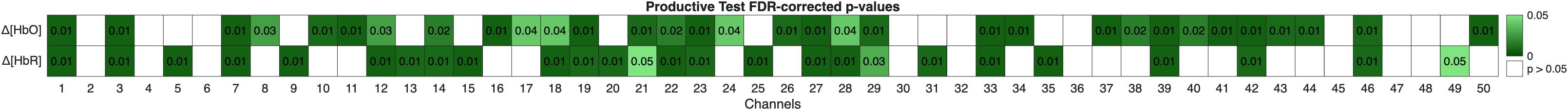

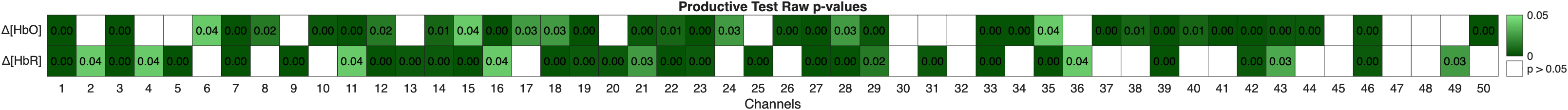
**Channel-wise comparison of raw and FDR-corrected p-values for the Productive Test condition.**

The top panel presents the raw p-values, and the bottom panel shows the Benjamini–Hochberg false discovery rate (FDR)-corrected p-values (q-values) for between-group comparisons (VR vs. Traditional) across all 50 fNIRS channels during the productive posttest. Results are shown for Δ[HbO] (upper row) and Δ[HbR] (lower row).

Each column corresponds to a channel, with darker green indicating stronger statistical significance (lower p- or q-values). Channels exceeding the significance threshold (p or q ≥ 0.05) are displayed in light gray, while missing channels are shown in white.

In the raw analysis, a large number of channels demonstrated significant group differences (HbO: 35/50; HbR: 32/50). Following FDR correction, a substantial proportion of these channels remained significant (HbO: 32/50; HbR: 26/50), confirming that many of the observed effects are robust to multiple-comparison correction.

Compared to the receptive condition, the productive test exhibits a stronger preservation of significant channels after FDR correction, particularly for HbO, suggesting that productive language tasks elicit more robust and reliable neural differences between learning modalities.
